# Cold atmospheric helium plasma causes synergistic enhancement in cell death with hyperthermia and an additive enhancement with radiation

**DOI:** 10.1038/s41598-017-11877-8

**Published:** 2017-09-15

**Authors:** Rohan Moniruzzaman, Mati Ur Rehman, Qing-Li Zhao, Paras Jawaid, Keigo Takeda, Kenji Ishikawa, Masaru Hori, Kei Tomihara, Kyo Noguchi, Takashi Kondo, Makoto Noguchi

**Affiliations:** 1 Department of Oral and Maxillofacial Surgery, Graduate School of Medicine and Pharmaceutical Sciences, University of Toyama Sugitani 2630, Toyama, 930-0194 Japan; 2 Department of Radiology, Graduate School of Medicine and Pharmaceutical Sciences, University of Toyama Sugitani 2630, Toyama, 930-0194 Japan; 3 Institute of Innovation for Future Society, Nagoya University, Furo-cho, Chikusa-ku, Nagoya, 4648603 Japan

## Abstract

Cold atmospheric plasmas (CAPs) have been proposed as a novel therapeutic method for its anti-cancer potential. However, its biological effects in combination with other physical modalities remain elusive. Therefore, this study examined the effects of cold atmospheric helium plasma (He-CAP) in combination with hyperthermia (HT) 42 °C or radiation 5 Gy. Synergistic enhancement in the cell death with HT and an additive enhancement with radiation were observed following He-CAP treatment. The synergistic effects were accompanied by increased intracellular reactive oxygen species (ROS) production. Hydrogen peroxide (H_2_O_2_) and superoxide (O_2_^•–^) generation was increased immediately after He-CAP treatment, but fails to initiate cell death process. Interestingly, at late hour’s He-CAP-induced O_2_^•–^ generation subsides, however the combined treatment showed sustained increased intracellular O_2_^•–^ level, and enhanced cell death than either treatment alone. He-CAP caused marked induction of ROS in the aqueous medium, but He-CAP-induced ROS seems insufficient or not completely incorporated intra-cellularly to activate cell death machinery. The observed synergistic effects were due to the HT effects on membrane fluidity which facilitate the incorporation of He-CAP-induced ROS into the cells, thus results in the enhanced cancer cell death following combined treatment. These findings would be helpful when establishing a therapeutic strategy for CAP in combination with HT or radiation.

## Introduction

Cancer is still the leading cause of deaths worldwide, with increasing incidence because of changing lifestyle and increased exposure to carcinogens^1^. Most of the available treatments like surgery, chemotherapy, radiotherapy are associated with undesirable side effects. Recent advancements in cancer biology led to the development of new methods to fight cancer and provided better insight into the molecular mechanisms of different cancers. Despite this, therapy resistance and non-selectivity are the main issues associated with the currently available treatments^2, 3^. Therefore, search for more selective anti-cancer strategy should be urgently required.

Plasma medicine is an emerging interdisciplinary field; plasma stated as the “fourth state of matter,” is a partially neutral an ionized gas, containing mixture of electrons, photons, atoms, positive and negative ions, radicals, various excited and non-excited molecules^4^. Cold atmospheric plasma (CAP) is an ionized low temperature gas, produced by applying a high voltage electric field at normal or atmospheric pressure. Recently, biomedical applications of CAP have gained great attention because of its promising potential applications such as sterilization^5, 6^, wound healing^7^ or blood coagulation^8^, dentistry^9^ and tissue regeneration^10^. However, the most increasingly important focus of CAP research is on the development of new therapeutic approaches based on its anti-cancer potential. Several studies have documented the efficacy of CAP for cancer treatment at both *in vitro* and *in vivo* experiments^11–15^. Although these demonstrated abilities were achieved by different plasma devices with difference in plasma properties, all studies showed the crucial role of reactive oxygen species (ROS) in plasma induced-anti-cancer effects^16^. The most distinctive feature of CAP application is the ability to selectively kill cancer cells, while sparing healthy cells. There is growing evidence that these selective anti-cancer effects are due to CAP-induced ROS and RONS in air and liquid environment^17^. Although, the cancer cells are particularly sensitive to ROS, however in the real clinical situation, it is very hard to treat cancer with single modality. The complete eradication of tumour cells is usually limited because of biological and technical problems. Therefore, a multimodality therapeutic strategy is adopted in which combination of physical therapy, as well as chemotherapeutics and certain agents which enhance the therapeutic effects of physical therapy were used.

It was recently shown that the synergistic effects of CAP in combination with nanoparticles and drugs have been highly regarded^18, 19^. The effects of CAP on other physical modalities such as hyperthermia (HT) and radiation has not been studied yet. Both HT and radiation are known anti-cancer therapies, the impact of HT and radiation alone or in combination have been well documented^20^. However, both therapies have been associated with un-intended effects because of exposure to high temperatures and radiation doses. Therefore, in this study the effects of helium cold atmospheric plasma (He-CAP) were investigated on HT 42 °C or low dose radiation 5 Gy and described the molecular insight involved in the combined treatment using human myelomonocytic lymphoma U937 cells.

## Results

### Synergistic enhancement of cell death following combined treatment with He-CAP and HT

**Figure 1:**
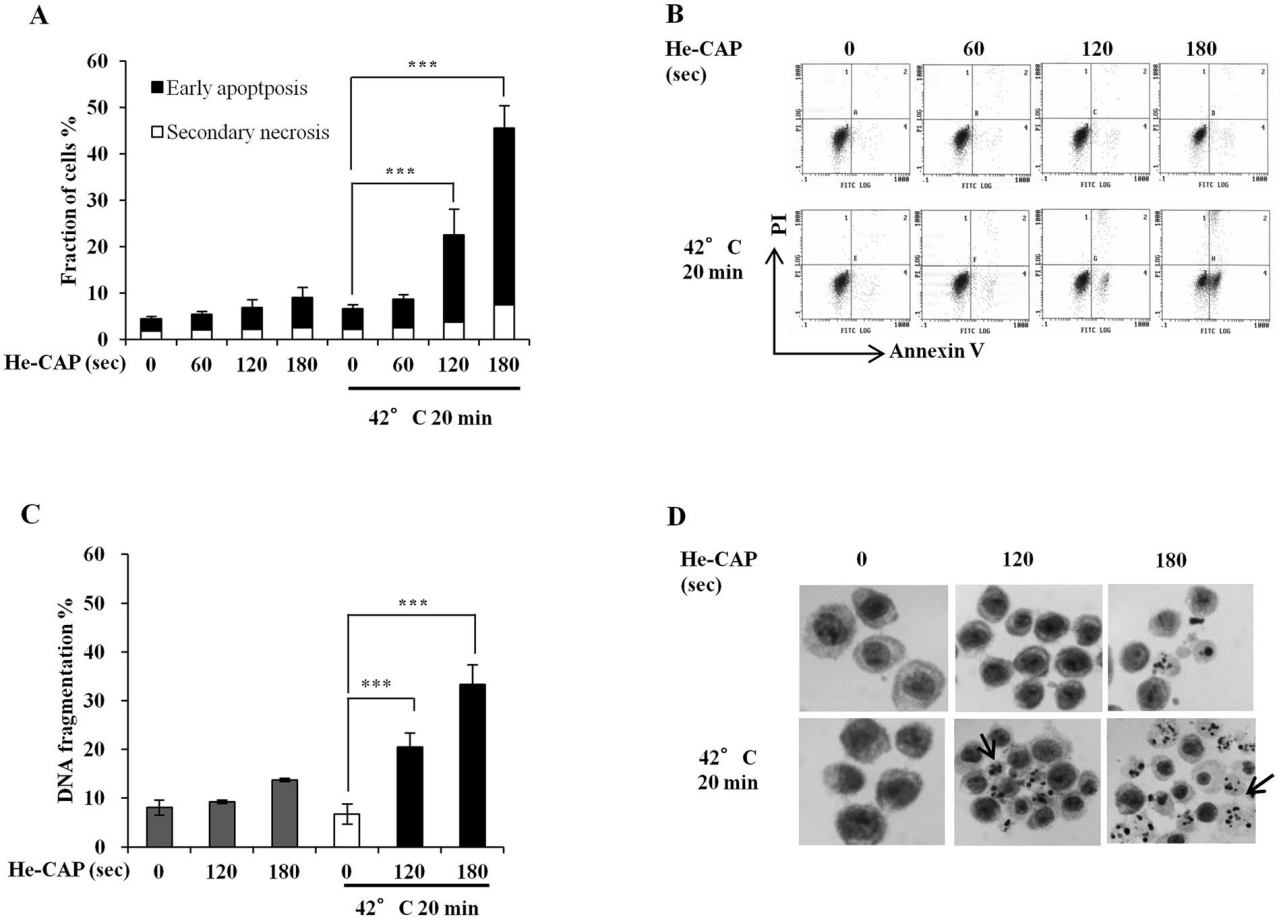
He-CAP and HT induced synergistic effects. (**A**) Annexin-V FITC/PI assay carried at 6 h after combined treatment with He-CAP and HT (n = 4). (**B**) Representative flow cytometry histograms of Annexin V-FITC/PI staining are shown. (**C**) Cells were harvested 6 h after combined treatment and subjected to DNA fragmentation assay (n = 3). (**D**) He-CAP and HT induced morphological features were detected by Giemsa staining. Images were taken at ×400 magnification under microscope. One representative photomicrograph is shown here, arrow head shows apoptotic cells. All the data are presented as mean ± SD. ***p < 0.005, *vs*. HT alone.

U937 cells were treated with He-CAP for 60 s, 120 s and 180 s, and exposed to HT at 42 °C for 20 min. After 6 h of post-treatment incubation, cells were subjected to annexin V-FITC/PI double staining. The results showed that the percentage of apoptotic cells induced by He-CAP and HT treatment alone were less than 10%, when cells were exposed to combined treatment; it was increased to 22.5% and 45.5% with 120 s and 180 s, respectively. However, no enhancement was observed with 60 s in combination with HT (Fig. 1A,B). Based on the findings, doses of He-CAP 120 s and 180 s were selected for exposure in the subsequent experiments. We also examined the effects of combined treatment on cell death by DNA fragmentation, a marked increase in the percentage of DNA fragmentation was observed following combined treatment compared to HT treatment alone (Fig. 1C). In addition, Giemsa staining showed that typical morphological features associated with apoptosis were more prominent in the combined treatment than either treatment alone (Fig. 1D). The efficacy of combined treatment was also evaluated at longer time period; cell survival was assayed by CCk-8 following combined treatment at 24 h, as shown in Supplementary Fig. S1A, the cell survival after HT treatment alone was 94% ± 7.8, however with combined treatment it declined to 66% ± 6.7 and 16% ± 2.0, with He-CAP 120 s and 180 s, respectively, thus showing synergistic enhancement in the cell death. Similarly the percentage of apoptotic cells and morphological features of apoptosis were further enhanced in the combined treatment at 24 h Supplementary Fig. S1B,C. These findings suggest that HT sensitize U937 cells to He-CAP treatment.

Further, the effects of this combination treatment were confirmed using human keratinocytes (HaCaT) cell line. HaCaT cells were treated with He-CAP for 120 s and 180 s, and then exposed to HT at 42 °C for 60 min. Cells were harvested following 24 h post-incubation and analyzed by annexin V-FITC/PI double staining. It was found that the number of early apoptotic cells with He-CAP 180 s slightly increased to 12.5% ± 3.4, and was less than 10% with He-CAP 120 s and HT treatment alone. No significant enhancement in the percentage of early apoptotic cells was observed following combined treatment as compared to He-CAP treatment alone. In the combined treatment early apoptosis increased in a similar extent as observed with He-CAP alone. However, the percentage of early apoptosis was increased with He-CAP alone and in combined treatment than HT treatment alone Supplementary Fig. S3A,B. This findings suggest that the combined treatment can selectively enhanced cell death in cancerous cells, while does not induced any apparent toxic effects in normal healthy cells.

### He-CAP and HT-induced intracellular ROS generation

Plasma irradiation has been known to induce immense quantities of free radicals. Electron paramagnetic resonance (EPR) spin trapping was performed with DMPO as a spin trap to detect the •OH radical generation after He-CAP exposure for 15 s to 60 s in aqueous solution at a distance of 2 cm from the tip of the plasma jet tube to the solution surface. The EPR signal ratio was increased following He-CAP exposure dose dependently; at 15 s 0.1 ± 0.0, 30 s 0.4 ± 0.1, 45 s 0.9 ± 0.5, and at 60 s it was 1.4 ± 0.7. Furthermore, the chemical activity of He-CAP was also confirmed by Fricke dosimetry following 60 s and found to be 0.6303 ± 0.02.

**Figure 2:**
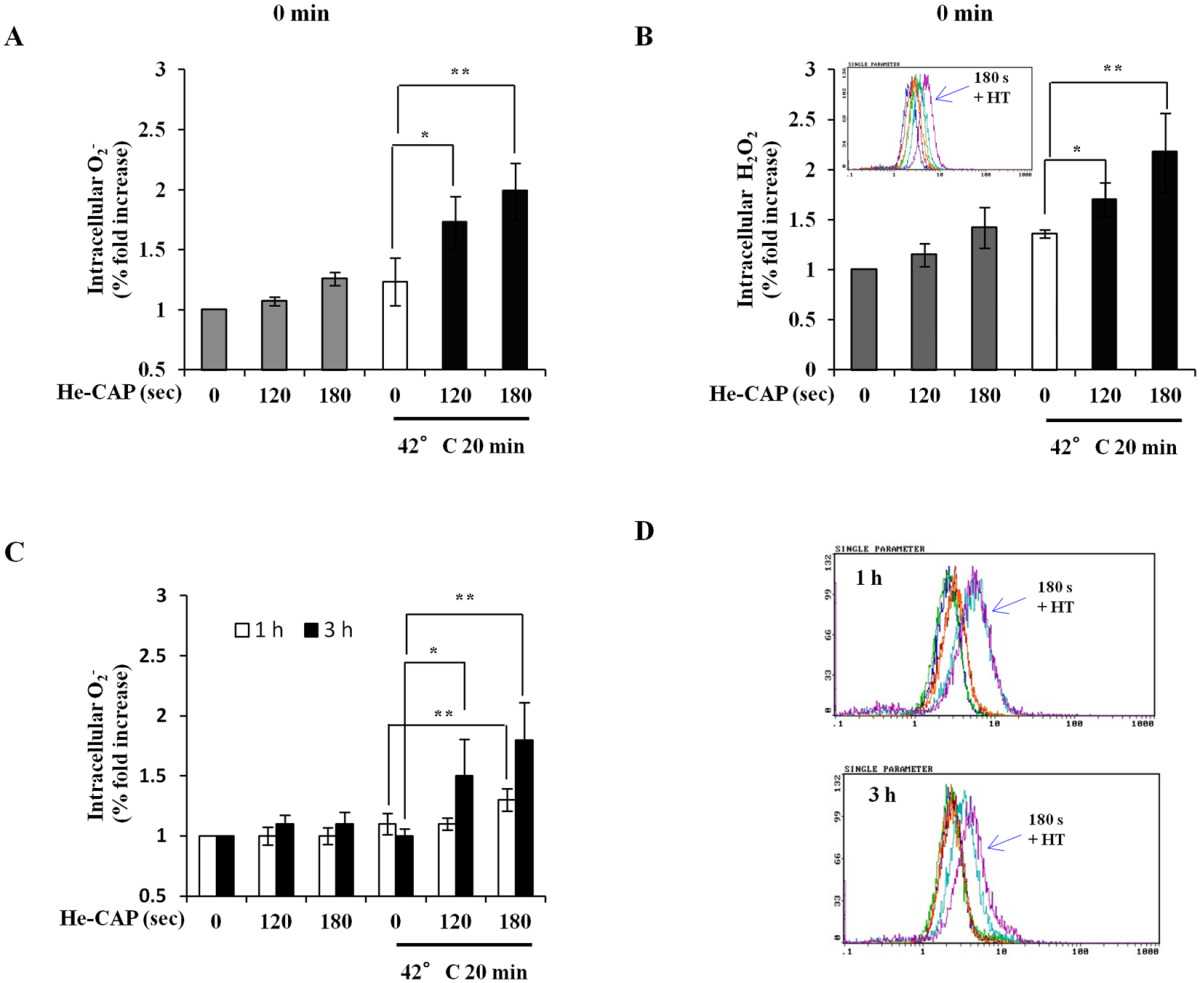
Intracellular ROS generation with He-CAP and HT combined treatment. Cells were harvested immediately after combined treatment or at indicated time. Elevated level of ROS species were analyzed by flow cytometry. (**A**) HE staining immediately after combined treatment. (**B**) DCFH-DA staining immediately after combined treatment. Upper small panel shows the representative histogram of DCFH-DA staining. (**C**) HE staining at 1 and 3 h following combined treatment. (**D**) Representative histogram of HE staining at 1 and 3 h are shown. Data are presented as the mean ± SD (n = 3). *p < 0.05, **p < 0.01, than the HT alone.

The involvement of ROS generation in the process was detected using two different ROS specific probes hydroethidine (HE) and Dichlorofluorescein diacetate (DCFH-DA). The superoxide (O_2_^•–^) generation measured immediately after treatment was increase following He-CAP and HT treatment alone and was markedly increased in the combined treatment (Fig. 2A). Similarly, the generation of Hydrogen peroxide (H_2_O_2_) was also increase immediately after either treatment alone, which was further substantially enhanced in the combined treatment (Fig. 2B). The intracellular detection of O_2_^•–^ generation at late hours showed that at 1 h and 3 h He-CAP and HT induced O_2_^•–^ generation subsides, however it remains strikingly elevated in the combined treatment. At 3 h more profound increased was observed in combination with He-CAP 120 s and 180 s than HT alone, at 1 h combination of He-CAP 180 s showed marked increased, while no change was observed in combination with He-CAP 120 s than HT alone (Fig. 2C,D). These findings showed that the intracellular ROS generation following combined treatment plays a crucial role in the synergistic enhancement of apoptosis induction.

### Effects of He-CAP and HT on mitochondrial membrane potential (MMP), intracellular [Ca^2+^]_i_, and ER stress signaling

**Figure 3:**
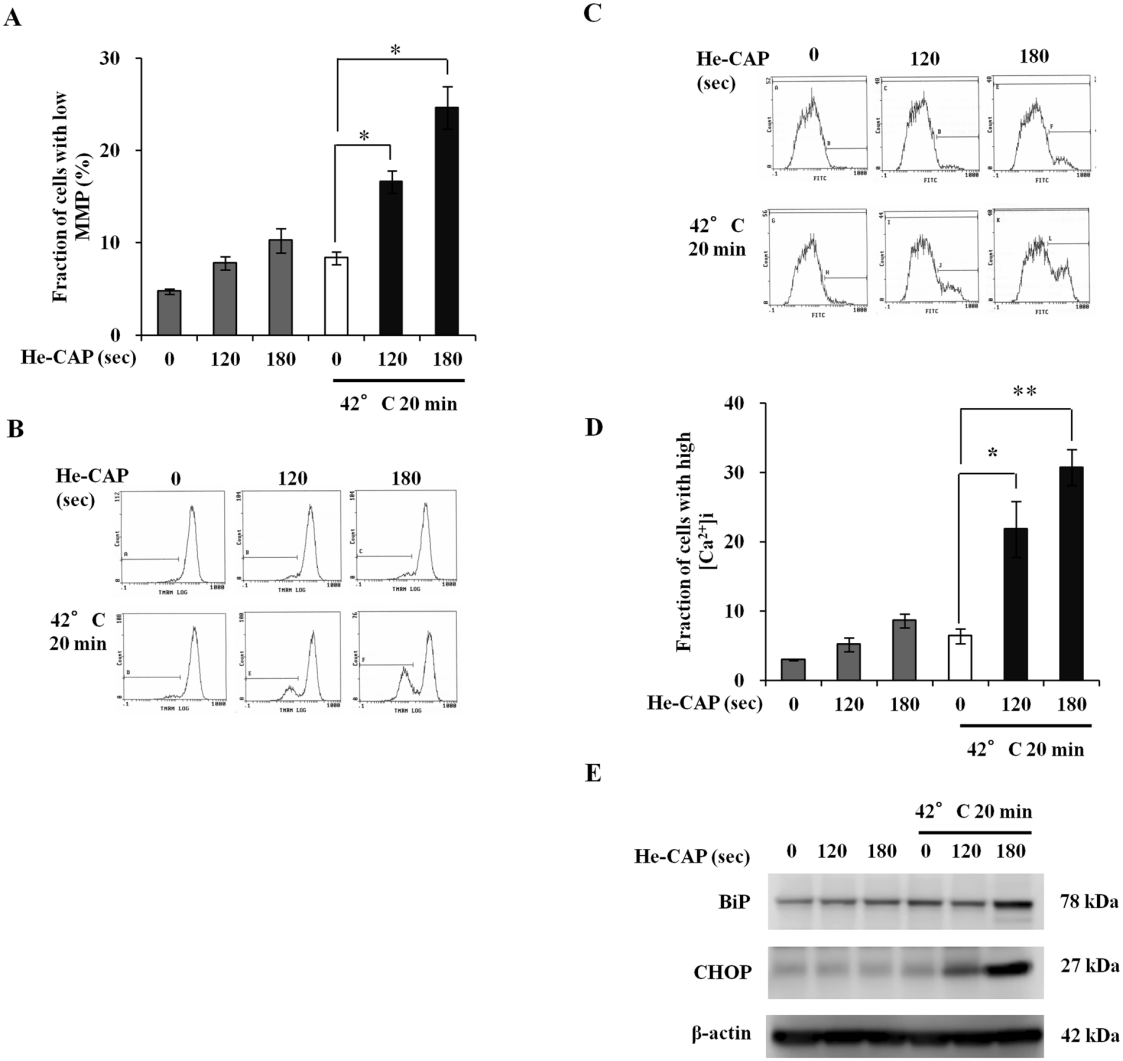
. He-CAP and HT combined treatment induced MMP loss, intracellular calcium release and ER-stress. (**A**) MMP loss was increased following combined treatment compared to either treatment alone (n = 3). (**B**) Representative flow cytometric histogram of MMP loss. (**C**) Representative flow cytometric histogram of [Ca^2+^]_i_ following combined treatment of He-CAP and HT. (**D**) [Ca^2+^]_i_ release was determined 6 h post-treatment, increased [Ca^2+^]_i_ was observed in the combined treatment (n = 4). Data are presented as the mean ± SEM. *p < 0.05, **p < 0.01, compared to HT alone. (**E**) Expression of ER-stress related proteins BiP and CHOP. β-actin was used to normalize the expression level in each sample. Blots were cropped, full lengths blots are presented in Supplementary Fig. 4.

To investigate the involvement of mitochondrial function in the enhancement of apoptosis, effects on the MMP were evaluated 6 h after combined treatment. The results showed that MMP loss, which is the end point of apoptosis, was not increased with either treatment alone. However, it was notably increased following combined treatment (Fig. 3A,B). The effects of combined treatment on intracellular calcium homeostasis were also examined. It was found that [Ca^2+^]_i_ concentration was markedly higher in the combined treatment than that in either treatment alone (Fig. 3C,D). To investigate the rationale for this [Ca^2+^]_i_ release_,_ the effects of combined treatment on ER were evaluated, as it contains the higher concentration of Ca^2+^. The downstream signaling of ER stress is mainly regulated through Bip/GRP78 and CHOP/GADD153, both are considered as the main regulator of ER-stress and their activation is the major indication for ER stress-induced cell death. The expression of Bip and CHOP markedly increased with combined treatment than in He-CAP and HT alone (Fig. 3E). These findings suggest the ROS-mediated activation of mitochondrial and Ca^2+^ dependent apoptotic pathway, and involvement of ER stress in it.

### Effects of He-CAP and HT on cell cycle distribution

**Figure 4:**
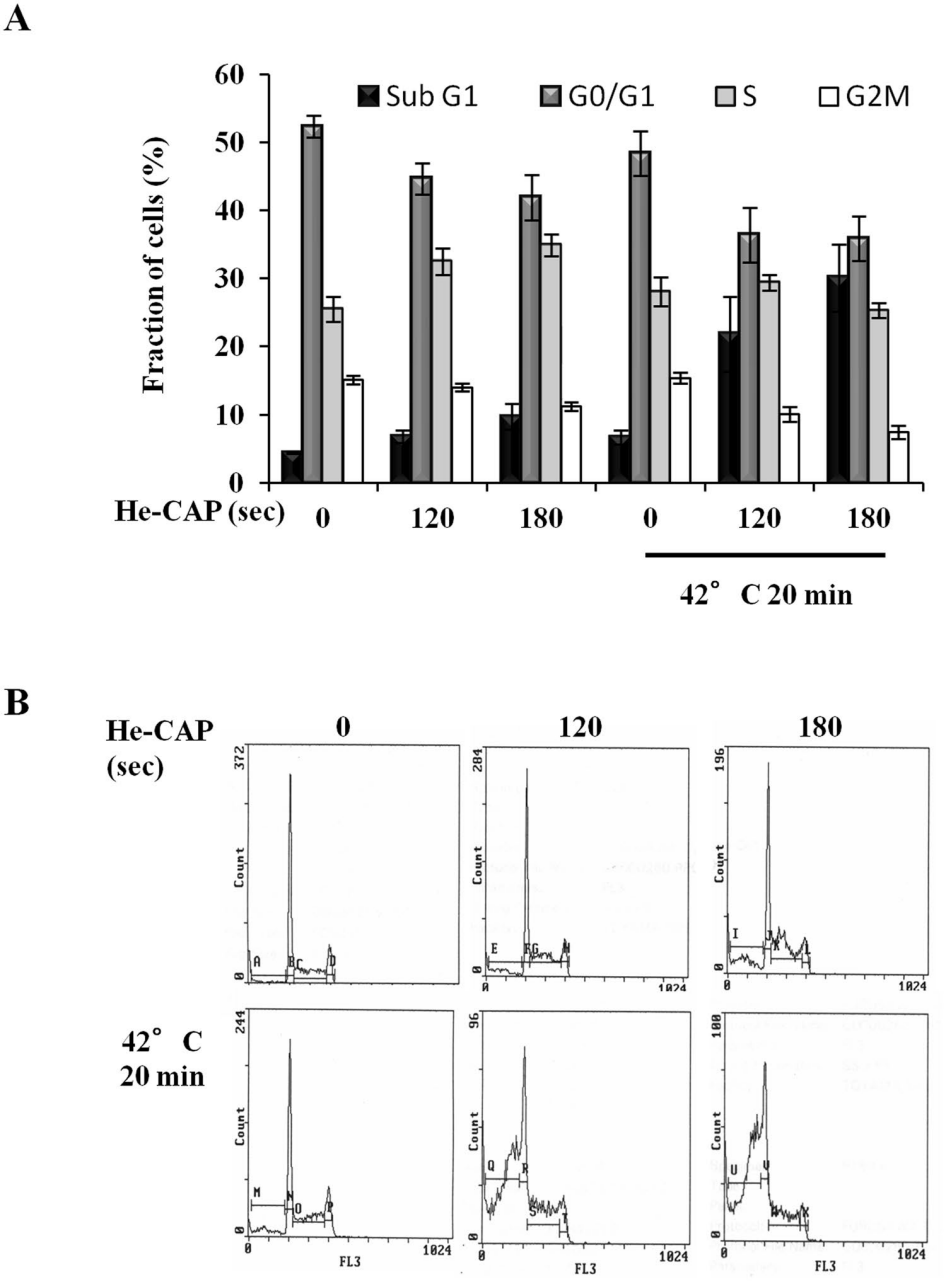
Cell cycle distribution following He-CAP and HT treatment. (**A**) Effect on cell cycle analysis was detected 6 h following combined treatment with He-CAP and HT. Increased fraction of cells in Sub G1 was observed. Data are presented as the mean ± SEM (n = 3). (**B**) One representative flow cytometric histogram of cell cycle analysis is shown.

The changes in cell cycle distribution induced by either treatment alone or in combination were shown in (Fig. 4A,B). He-CAP treatment alone showed slight increase in the fraction of S phase cells. He-CAP or HT treatment alone does not show marked increase in the percentage of sub G1 fraction cells. However, in the combined treatment the percentage of sub G1 fraction was markedly increased to 21.86 ± 5.5 S.E.M and 30.07 ± 4.9 S.E.M, than either treatment alone. This increased in the sub G1 fraction was brought out with decrease in G1 and G2/M phases, following combined treatment, which is caused by the induction of apoptosis.

### Expression of apoptosis-related proteins

**Figure 5:**
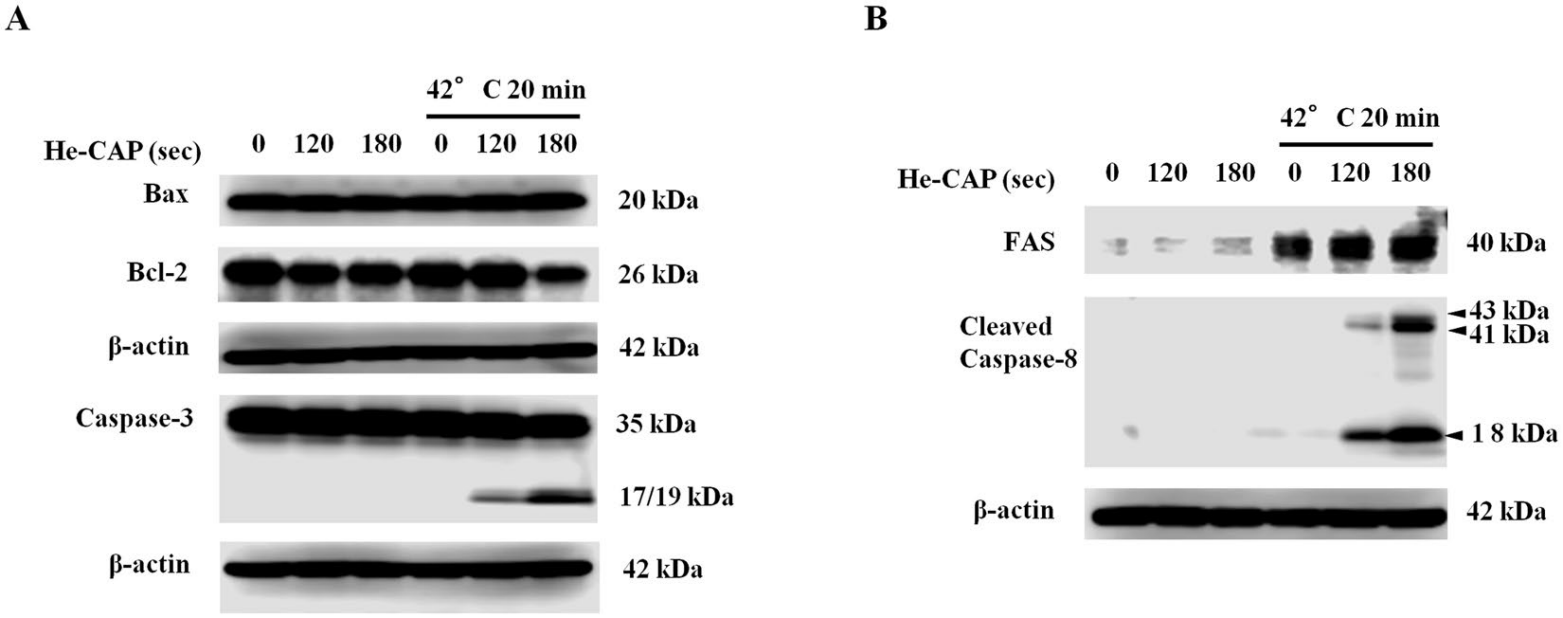
He-CAP and HT induced activation of apoptosis related proteins. Cells were harvested 6 h post-treatment following combined treatment with He-CAP and HT and subjected to Western blot (**A**) Expression of Bcl-2 family proteins Bax, Bcl-2 and activation of caspase-3. (**B**) Changes in the expression of FAS and caspase-8 activation. β-actin was used to normalize the expression level in each sample. Cropped blots are shown, full lengths blots are presented in Supplementary Figs 5, and 6.

Bcl-2 family proteins with anti- or pro-apoptotic functions are responsible for mitochondrial transmembrane permeability and release of cytochrome c, to activate caspase cascade. The expression of anti-apoptotic Bcl-2 was decreased after combined treatment with He-CAP 180 s and HT, while the expression of pro-apoptotic Bax was remained unchanged (Fig. 5A). In addition, the combined treatment induced effects on caspases, which are the main executioner of apoptosis were evaluated. The active form of caspase-3 (cleaved capase-3) was markedly increased in the combined treatment than in He-CAP and HT treatment alone (Fig. 5A).

### FAS externalization and caspase-8 activation induced by combined treatment

The activation of FAS receptor is linked to the initiation of extrinsic pathway of apoptosis, *via* a DISC assembly and subsequent caspase-8 activation. The results showed that FAS protein expression was not observed with He-CAP treatment alone, while HT treatment showed slight increased Fas expression. The expression of FAS was markedly increased in the combined treatment. Simultaneously, caspase-8 activation was also only evident in the combined treatment, with no expression in either treatment alone (Fig. 5B).

### He-CAP and HT combined treatment induces synergistic induction of apoptosis in other cancer cells irrelevant to p53 status

The effects of He-CAP and HT in MOLT-4 (wild type p53) and HCT-116 (wild type p53) were also studied. MOLT-4 cells showed sensitivity towards He-CAP treatment. The percentage of early apoptotic (annexin V-positive and PI-negative) cells in He-CAP 60 s alone was 18.9 ± 6.0, while after the combined treatment the number of early apoptotic cells markedly increased to 35.8 ± 6.8, no significant change was observed in the percentage of late apoptotic (annexin V-positive and PI-positive) cells, Supplementary Fig. S2A,B. In addition, cell survival percentage also decreased to 52 ± 4.8 in the combined treatment, which was 85 ± 5.6 with He-CAP 60 s, Supplementary Fig. S2C. In HCT-116 cells the number of early apoptotic and late apoptotic cells increased to 22.1 ± 3.2 and 32.2 ± 1.73 with combined treatment of HT 42 °C 60 min and He-CAP 120 s, 180 s, than either treatment alone after 24 h Supplementary Fig. S2D,E. These findings suggested that the combination of He-CAP and HT induced synergistic cell death independent to p53 mutations.

### Effects of He-CAP on radiation-induced cell death in U937 cells

**Figure 6:**
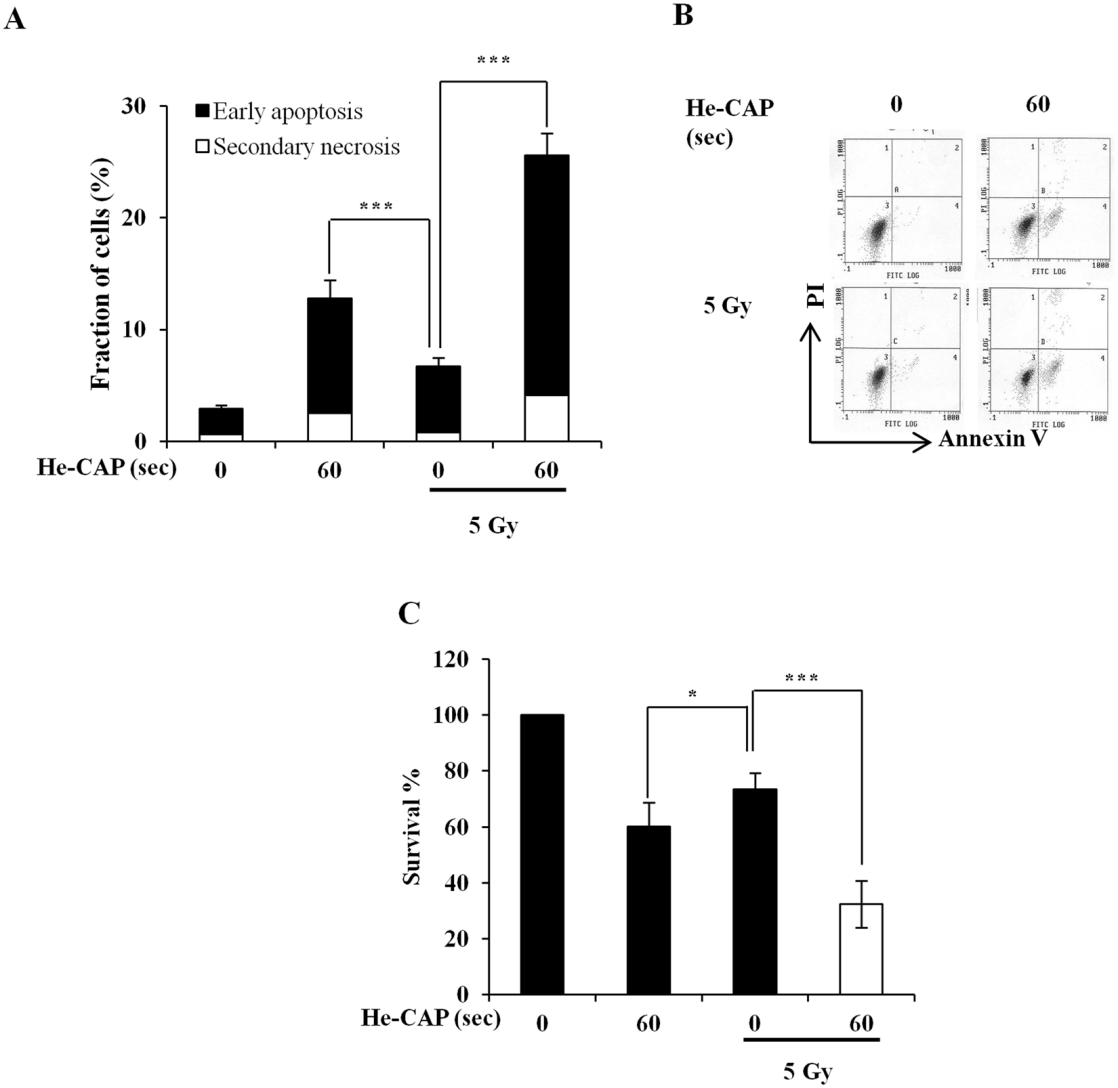
He-CAP and radiation-induced cell death in U937 cells. (**A**) Annexin V-FITC/PI assay following treatment of He-CAP 60 s and radiation 5 Gy. (**B**) Representative flow cytometric histogram of Annexin V-FITC/PI staining with He-CAP and radiation. (**C**) Cell survival assay by cell counting kit-8 was carried at 6 h post-treatment. Data are presented as the mean ± SD (n = 4). *p < 0.05, ***p < 0.005, *vs*. treatment alone.

The combined effects of He-CAP 60 s and radiation (5 Gy) were evaluated by exposing 0.1 × 10^6^ cells/ml. The results showed that He-CAP treatment 60 s alone induced marked apoptosis as compared to radiation alone. Although, the combined treatment resulted in the enhanced apoptosis but the overall increased in cell death was only additive enhancement (Fig. 6A,B). Similarly, cell survival percent after treatment with He-CAP 60 s decreased to 60% ± 8.5, with radiation it was 73 ± 5.7% and following combined treatment substantially decreased to 32% ± 8.3. These findings also showed the additive effects after combined treatment (Fig. 6C).

## Discussion

CAP is a potential source of active agents, and mounting evidence suggests that the effects of CAP are mainly mediated *via* generation of ROS and lead to apoptosis^21^, cellular necrosis^22^, and senescence^23^. The most appealing feature of CAP, it’s selectively against cancer cells is dependent on the different basal intracellular ROS level in cancer and normal cells. Cancer cells tend to posses the higher metabolism and basal ROS level than the normal cells, which make them more susceptible to exogenous ROS stress and ultimately lead to initiation of apoptosis or cell death^24, 25^. However, one of main hindrance in the development of CAP device for clinical application is lack of standardization in between CAP devices because the anti-cancer activity of CAP is directly linked with its ability to produce ROS and RONS, which can enormously vary in between CAP devices^26, 27^. The properties of CAP can be modified depending on experimental conditions such as plasma setup, voltage applied, feeding gas, gas flow rate, distance from the plasma source and volume of solution, etc^28, 29^. Despite these variances in the effects of CAP devices, one common aspect among all CAP models is the generation of ROS and RONS. A previous review suggested the selective anti-cancer capacity of CAP based on model of aquaporins (AQPS), they proposed that cancer cells express more AQPS, which made them particularly sensitive to CAP-induce ROS than normal cells.^30^ These findings are not in agreement with other published studies which showed that not all AQPS are able to transport H_2_O_2_ efficiently across the membrane^31^, and the effects of CAP treatment were common to both normal and cancer cells, even cancer cells are more resistant than normal cells^17, 32, 33^. We previously demonstrated that argon-cold atmospheric plasma (Ar-CAP) can induce higher levels of hydroxyl (•OH) radicals in an aqueous solution i-e approximately 30 times the amount of •OH radicals produced by X-irradiation. However, the apoptosis inducing ability of X-irradiation remains superior to Ar-CAP irradiation^34^. These findings highlight the problem of limited interaction and penetration of CAP-induced extracellular ROS through plasma membrane. To affect cancer cells CAP-generated ROS and RONS in the liquid phase, must be incorporated through the plasma membrane or react with plasma membrane to induce intracellular ROS through lipid peroxidation^35, 36^.

For clinical application of CAP it is necessary to develop one standardized therapeutic strategy based on common aspect of CAP models. Therefore, in this study we have demonstrated a useful strategy by combining He-CAP with HT and radiation, in which HT or radiation facilitates the incorporation of CAP-induced extracellular ROS inside the cells and enhances its efficacy. HT and radiation, alone or in combination with chemotherapy have shown promising anti-cancer effects for various cancer and the effects of these combination therapies have been verified in a clinical trial^37^^.^ He-CAP in combination with HT causes a synergistic enhancement in apoptosis. The He-CAP induced •OH formation in an aqueous solution was observed, based on the quantification of electron paramagnetic (EPR) spectra. Similarly, the intracellular ROS formation in U937 cells with He-CAP treatment was increased as detected by DCFH-DA and HE staining (Fig. 2). Despite initial increased in the He-CAP-induced ROS formation, apoptosis induction was not observed (Fig. 1). However, in contrast, following combination with HT apoptosis was synergistically enhanced and well corresponds to the intracellular ROS generation levels (Fig. 2). Based on our data it is important to note that although CAP can stimulate the generation of intracellular ROS, but it’s for shorter time period and below threshold to activate the apoptotic machinery. In comparison, CAP-induced enormous amount of ROS in the liquid phase, therefore the enhancement of cell death is mainly attributed due to the plasma-delivered ROS from outside to inside. The possible mechanism involved in this synergistic enhancement is because of HT-induced changes on the cancer cell membrane. The heat stress causes disruptions of cytoskeleton structures like microtubules and microfilaments, which lead to the disorganized organelle localization and the breakdown of intracellular transport process. In addition, HT can affects membrane fluidity and fragility, during heating alter membrane permeability towards several compounds have been observed including anti-cancer drugs^38^. Therefore, it was speculated that HT treatment facilitates the incorporation of He-CAP-induced ROS into the cells. Once this intracellular ROS exceeds beyond the threshold level, it caused enhanced cell death following combined treatment. This notion was supported by a finding that at late hours He-CAP and HT inducedO_2_^•–^ generation was subside, but it remains elevated in the combined treatment (Fig. 2C). This suggests that incorporation of ROS results in the activation of apoptotic machinery as sustained elevation of O_2_^•–^ is believed to be due to the xanthine oxidase activation and/or mitochondria respiratory reaction chain^39^. Consistent with our findings, recent studies also showed that the transmembrane diffusion of CAP-induced ROS does not occur freely. High energy barriers prevent the entry of ROS through the oxidized phopsholipid bilayer. The delivery of ROS into the cell interior requires porous membrane structural changes, which can be achieved by applying electric field^40^, nanoparticles^41^, and due to the effects of cholesterol on permeation *via* lipid bilayer^42^. Intracellular oxidative stress induced by ROS plays a crucial role in the apoptosis induction *via* both intrinsic (mitochondrial) and extrinsic (death receptor) pathway^43, 44^. Our results showed the involvement of intrinsic pathway as mitochondrial membrane disruption, increase in intracellular calcium and expression of ER stress marker Bip and CHOP was increased following combined treatment than the either treatment alone (Fig. 3). The expression of anti-apoptotic Bcl-2 protein was decreased with combined treatment of He-CAP 180 s and HT, unfortunately no effect was observed on the pro-apoptotic Bax expression in total cell lysates. Furthermore, up-regulation of FAS-receptor was also observed following combined treatment. FAS (CD95) has been regarded as the prototypic and major member of death receptor family, its activation is associated with ROS related apoptosis^45, 46^. The death receptors, especially FAS are the most abundant transmembrane receptors in the membrane raft domains^47, 48^. Disruptions of membrane fluidity and lipid rafts have been linked in the course of apoptosis. Heat shock and HT treatment increases membrane fluidity and can alter the membrane raft microdomains leading to the death receptor activation and apoptosis^49, 50^. Of note, we found that HT treatment alone increases FAS expression compared to He-CAP treatment alone. However, the activation of FAS downstream signaling caspase-8 was not observed with HT treatment alone. This finding suggests that HT initially induces FAS activation either due to the increase membrane fluidity or interaction of HT-induced intracellular ROS. Therefore, in the combined treatment further interaction of He-CAP-induced ROS with activated FAS triggers profound increase in the FAS activity and ultimately caused the activation of caspase-8 (Fig. 5B). FAS/TNF-RI can induce apoptosis *via* a direct recruitment of caspase cascade or *via* mitochondria by activating caspase-8 and Bid^51^. The two apoptotic ways could be interconnected by caspase-8 mediated cleavage of Bid, which leads to the activation of mitochondrial pathway (intrinsic), and ultimately leads to the activation of effector caspase (caspase-3).

We also determined the effects of He-CAP in combination with radiation. At first cells were treated with He-CAP and radiation maintaining the same treatment conditions as in case of HT. However, cell death was not observed. Considering the fact that effects of CAP or CAP activated medium are greatly influence by several factors including cell density^52^. We therefore examined the effects at lower density of 0.1 × 10 ^6^ cells/ml. It was expected that exposing cells at lower density would result in more profound synergistic effects following combined treatment with He-CAP (60 s) and radiation (5 Gy). However, in contrast, the combined treatment with He-CAP and radiation showed only additive enhancement in the apoptosis (Fig. 6). U937 cells at low density were exposed, He-CAP 60 s alone showed marked amount of apoptosis (Fig. 6A), which was not observed when U937 cells were exposed at a density of 1 × 10^6^ cells/ml (Fig. 1A). If it were the membrane fluidity or an alteration that justifies the synergistic enhancement in combination with HT, one might speculate that similar effect should be observed also with radiation. It is important to note that the degree of membrane fluidity induced by radiation is not same as HT and typical biological effects of radiation-induced cell death are mostly because of its indirect action^53^. The interaction of CAP in combination with radiation will need to be evaluated in future studies.

In summary, this study provides the initial piece of evidence regarding the combined used of CAP with other physical modalities. The synergistic enhancement in apoptosis with He-CAP and HT was not only confined to U937 cells, rather it was also observed in other cell lines harbouring different p53 status such as MOLT-4, and HCT-116 (see Supplementary Fig. S2). Interestingly, more profound synergistic effects were observed in U937, which are p53 mutant cells. Loss of functional p53 pathway is common in human’s tumors, which contributes to aggressive tumor behavior and therapeutic resistance^54^. These findings emphasize the efficiency of combined treatment with HT, as synergistic effects were achieved when cancer cells were exposed at higher densities, irrelevant to p53 status. We have demonstrated the strategy for possible future clinical application of CAP with HT or radiation. This plasma-thermia or plasma-hyperthermia strategy would help to overcome the barrier regarding CAP clinical application, such as limited penetration of ROS, variance in CAP devices and its induced effects.

## Material and Methods

### Cell culture

A human myelomonocytic lymphoma cell line, U937, MOLT-4, and human colon carcinoma cell line HCT-116 were obtained from Human Sciences Research Resource Bank (Japan Human Sciences Foundation, Tokyo, Japan). The human keratonicyte cell line (HaCaT) was obtained from Department of Oral and Maxillofacial surgery, which was kindly gifted by Dr T. Shimizu, Department of Dermatology, University of Toyama. The U937 and MOLT-4 cells were grown in RPMI 1640 culture medium. HaCaT cells were cultured in Dulbeccos modified Eagles medium (DMEM). HCT-116 cells were grown in McCoy’s 5a medium. All mediums were supplemented with 10% heat-inactivated fetal bovine serum (FBS). Cell cultures were maintained at 37 °C in humidified air with 5% CO_2_.

### Cold atmospheric helium plasma irradiation system

A cold atmospheric plasma system (PN-120TPG, NU Global, Nagoya, Japan) consisted of a gas flow controller, a voltage power supply and a hand-piece of the plasma jet, constructing an inner micro hollow-type electrode and an outer dielectric barrier electrode. The inner and outer diameter of dielectric tube was 1 and 2 mm respectively. A high-voltage power with a frequency of 60 Hz and a peak-to-peak voltage of 7 kV was supplied to the two electrodes. Helium gas with a gas flow rate of 2 L/min was applied in this study for the generation of a plasma jet. The line-averaged electron density in the plasma source is approximately 2 × 10^15^ cm^−3^. The length of the plasma jet was approximately 20 mm in atmospheric ambient. The gas temperature of the plasma jet was below 350 K.

### Electron paramagnetic resonance (EPR)-spin trapping for detection of hydroxyl radicals (•OH)

The detection of •OH radicals induced following exposure to He-CAP was carried out using the EPR-spin trapping with DMPO as a spin trap. An aqueous solution containing a spin trap at a concentration of 10 mM was irradiated at increasing duration from 15 s to 60 s. Immediately after He-CAP treatment, the samples were transferred to a glass capillary tube (VC-HO75P, Terumo, Tokyo, Japan) and inserted into a special quartz tube in a cavity of an EPR spectrometer (RFR-30, Radical Research Inc., Tokyo, Japan). In general, EPR setting were microwave power; 4 mW, frequency; 9.425 GHz, center magnetic field; 329.5 mT, and modulation width; 0.1 mT. The EPR spectra of the treated samples were recorded at room temperature.

### Chemical dosimeter

The chemical effects of He-CAP were measured by a ferrous-ferric ion (Fricke) dosimeter. Changes in absorbance of the chemical system with exposure time were determined with a spectrophotometer at 304 nm.

### Cell treatments

U937 and MOLT-4 cells were cultured in a 24 well plate with 1 ml of RPMI1640, HaCaT and HCT-116 cells were cultured in a 24 well plate with 1 ml of DMEM and McCoy’s 5a medium, respectively. Cells were treated to He-CAP at a distance of 2 cm from the tip of plasma jet tube to the solution surface. For hyperthermia treatment, after He-CAP treatment, 1 × 10^6^/ml U937 and MOLT-4 cells were transferred to plastic tubes, and exposed to HT at 42 °C for 20 min by immersing tubes containing cell suspension into a precision-controlled water bath. For HaCaT and HCT-116 cells, 24 well plates were sealed with paraffin film and placed in water bath at 42 °C for 60 min.

For radiation, after He-CAP treatment, cells were irradiated at room temperature at a dose of 5 Gy using the X-ray generator (MBR-1520R-3, Hitachi Medical Technology Co., Kashiwa, Japan) operating at 150 kV and 20 mA at a dose rate of 5 Gy/min as determined by Fricke dosimetry. After the treatment, cells were incubated at 37 °C and were harvested at the indicated time periods.

### Detection of apoptosis using Annexin V-FITC/ PI staining

Flow cytometry was performed with propidium iodide (PI) and fluorescein isothiocyanate (FITC)-labeled annexin V (Immunotech, Marseille, France) to detect phosphatidylserine externalization. After the treatments, cells were incubated at 37 °C for 6 or 24 h, collected, washed with PBS and centrifuged at 1200 rpm for 3 min. The resulting pellet was mixed with the binding buffer of the Annexin V-FITC kit. FITC-labeled Annexin V (5 μl) and PI (5 μl) were added to 490 μl of cell suspension and mixed gently. After incubation at 4 °C for 30 min in the dark, the cells were analyzed by flow cytometry (Epics XL, Beckman-Coulter, Miami, FL).

### DNA fragmentation assay

Quantitative DNA fragmentation assay was carried out 6 h post-treatment using the method of Sellins and Cohen^55^, with minor modifications. Briefly, approximately 3 × 10^6^ cells were lysed using 200 μl of lysis buffer (10 mM Tris, 1 mM EDTA and 0.2% Triton X-100, pH 7.5) and centrifuged at 13,000 g for 10 min. Subsequently, each DNA sample in the supernatant and the resulting pellet was precipitated in the 25% trichloroacetic acid (TCA) at 4 °C overnight and quantified using a diphenylamine reagent after hydrolysis in 5% TCA at 90 °C for 20 min. The percentage of fragmented DNA in each sample was calculated as the amount of DNA in the supernatant divided by total DNA for that sample (supernatant plus pellet).

### Assessment of morphological changes

The morphological changes in the cells were examined by Giemsa staining. Cells were harvested after 6 or 24 h of post-incubation at 37 °C, washed with PBS and collected by centrifugation. Then the cells were fixed with methanol and acetic acid (3:1) and spread on the glass slides. After drying, staining was performed with 5% Giemsa solution (pH 6.8) for 5 min.

### Cell viability assay

Cell viability was determined using the colorimetric cell counting kit-8 assay (CCK-8; Dojindo Laboratories Co., Ltd., Kumamoto, Japan). Briefly, after 6 or 24 h post-treatment, cells were incubated in 100 μl RPMI medium (containing 10 μl CCK-8) in 96-well plate and then further incubated for 2 h at 37 °C in 5% CO_2_, according to the manufacturer’s instructions. Absorbance at 450 nm was detected by using Microplate Reader (Bio-Rad Laboratories, Inc. Hercules, CA, USA).

### Assessment of intracellular reactive oxygen species

Fluorescent probes differentially sensitive to different ROS were employed to detect the extent of change in intracellular oxidative stress in treated U937 cells following exposure to He-CAP and HT. DCFH-DA (Molecular probes, Eugene, OR) and HE (Molecular Probes) was used to determine H_2_O_2_ and O_2_^•–^, respectively. Briefly, cells were collected immediately or after post-treatment at indicated time points, washed with PBS, then DCFH-DA was added at final concentration of 10 μM and HE was added at final concentration of 5 μM. Cells were incubated for 15 min at 37 °C. The fraction of fluorescence positive cells was measured by flow cytometry as the proportion of cells containing intracellular ROS.

### Measurement of mitochondrial membrane potential

To measure changes in MMP, after the treatments cells were incubated at 37 °C for 6 h, collected, washed with PBS and stained with 10 nM tetra-methylrhodamine methyl ester (TMRM; Molecular Probes, Eugene, OR) for 15 min at 37 °C in 1 ml of PBS, followed by the immediate flow cytometry of red TMRM fluorescence (excitation at 488 nm; emission at 575 nm).

### Measurement of [Ca^2+^]_i_

The effects of combined treatment on intracellular calcium homeostasis, intracellular free Ca^2+^ was measured using calcium probe Fluo-3/AM (Dojindo Laboratories Co., Ltd., Kumamoto, Japan). After 6 h of post-treatment incubation at 37 °C, the cells were harvested and then loaded with 5 μM Fluo-3/AM for 30 min at 37 °C. Excess Fluo-3/AM was removed by washing three times with PBS. The fluorescence intensity of free Ca^2+^ levels was measured by flow cytometry.

### Cell cycle analysis

At 6 h following combined treatment, cells were fixed with pre-chilled 70% ice cold ethanol and stored overnight at −20 °C. Subsequently, fixed cells were treated with 0.25 mg/ml RNase A (Nacalai Tesque, Kyoto, Japan) and 50 μg/ml PI in PBS. The samples were finally run on an Epics XL flow cytometer (Beckman-Coulter, Miami, FL) to obtain the distribution of PI-based cell-cycle phases.

### Western blot analyses

The cells were collected and washed with cold PBS. Cells were lysed at a density of 2.0 × 10^6^ cells ⁄ 100 μl of RIPA buffer (50 mM Tris-HCl, 150 mM NaCl, 1% Nonidet P-40 (v⁄v), 1% sodium deoxycholate, 0.05% SDS, 1 μg of each aprotinin, pepstatin and leupeptin and 1 mM phenylmethyl sulfonyl fluoride) for 20 min. Following brief sonification, the lysates were centrifuged at 12,000 g for 10 min at 4 °C, and the protein content in the supernatant was measured using the Bio- Rad protein assay kit (Bio-Rad, Hercules, CA). Protein lysates were denatured at 96 °C for 5 min, after mixing with SDS-loading buffers, applied on an SDS-polyacrylamide gel (Daiichi Pure Chemicals Co., Ltd, Tokyo, Japan) for electrophoresis, and transferred to nitrocellulose membrane (Amersham Biosciences, Buchinghamshire, UK). Western blot analysis was performed to detect Caspase-3, Cleaved caspase-8, Bax, Bcl-2, Fas, Bip, CHOP and β-actin expression using specific polyclonal antibodies. Blots were then probed with either secondary horseradish peroxide (HRP)-conjugated anti-rabbit or anti-mouse IgG antibodies obtained from Cell Signaling. Band signals were visualized on a LI-COR image analyzer (Linclon, Nebraska, USA) by using either chemi-Lumi One L (Nacalai Tesque, Kyoto, Japan) or ImmunoStar LD (Wako, Japan) detection reagents.

### Statistical analysis

The values are expressed as the means ± standard deviation (SD) or standard error of the mean (SEM), where indicated. The statistical significance of difference was evaluated using the Student’s *t*-test. Values of p < 0.05 were considered to be significant. All experiments were performed at least in triplicate.

## Electronic supplementary material

Supplementary information
