# Supplementary material for: Cold atmospheric helium plasma causes synergistic enhancement in cell death with hyperthermia and an additive enhancement with radiation
Source: Sci Rep. 2017 Sep 15;7:11659. doi: 10.1038/s41598-017-11877-8 (PMC5600975; doi:10.1038/s41598-017-11877-8)
Supplement: Supplementary file 1 — Supplementary information [file 41598_2017_11877_MOESM1_ESM.pdf]

Cold atmospheric helium plasma causes synergistic enhancement in cell death with hyperthermia and an additive enhancement with radiation

Rohan Moniruzzaman [1, +], Mati Ur Rehman [2,*, +], Qing-Li Zhao [2], Paras Jawaid [2], Keigo Takeda [3], Kenji Ishikawa [3], Masaru Hori [3], Kei Tomihara [1], Kyo Noguchi [2], Takashi Kondo [2], Makoto Noguchi [1]

[1] Department of Oral and Maxillofacial Surgery, Graduate School of Medicine and Pharmaceutical Sciences, University of Toyama Sugitani 2630, Toyama, 930-0194, Japan.

[2] Department of Radiology, Graduate School of Medicine and Pharmaceutical Sciences, University of Toyama Sugitani 2630, Toyama, 930-0194, Japan.

[3] Institute of Innovation for Future Society, Nagoya University, Furo-cho, Chikusa-ku, Nagoya 4648603, Japan.

Figure 1. supplementary online

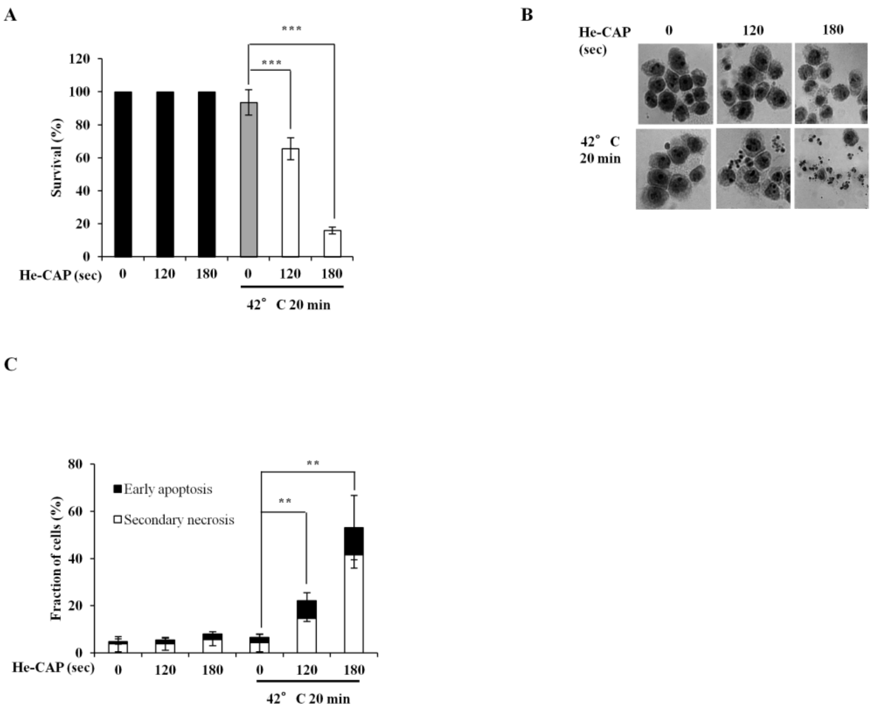

Fig. S1. He-CAP and mild HT induced synergistic effects were detected at 24 h. (A) Cell survival assay with cell counting kit-8 was carried out 24 h post-treatment after combined treatment with He-CAP and HT. Cell survival was significantly decreased with combined treatment of He-CAP 120 s and 180 s with mild HT. (B) The apoptotic features were further increased at 24 h as evident by Giemsa staining. (C) Evaluation of apoptosis at 24 h showed increased fraction of secondary apoptosis in the combined treatment. Data are shown as mean ± SD (n=3), **$p<0.01$, ***$p<0.005$, compared to HT treatment alone.

Figure 2. supplementary online

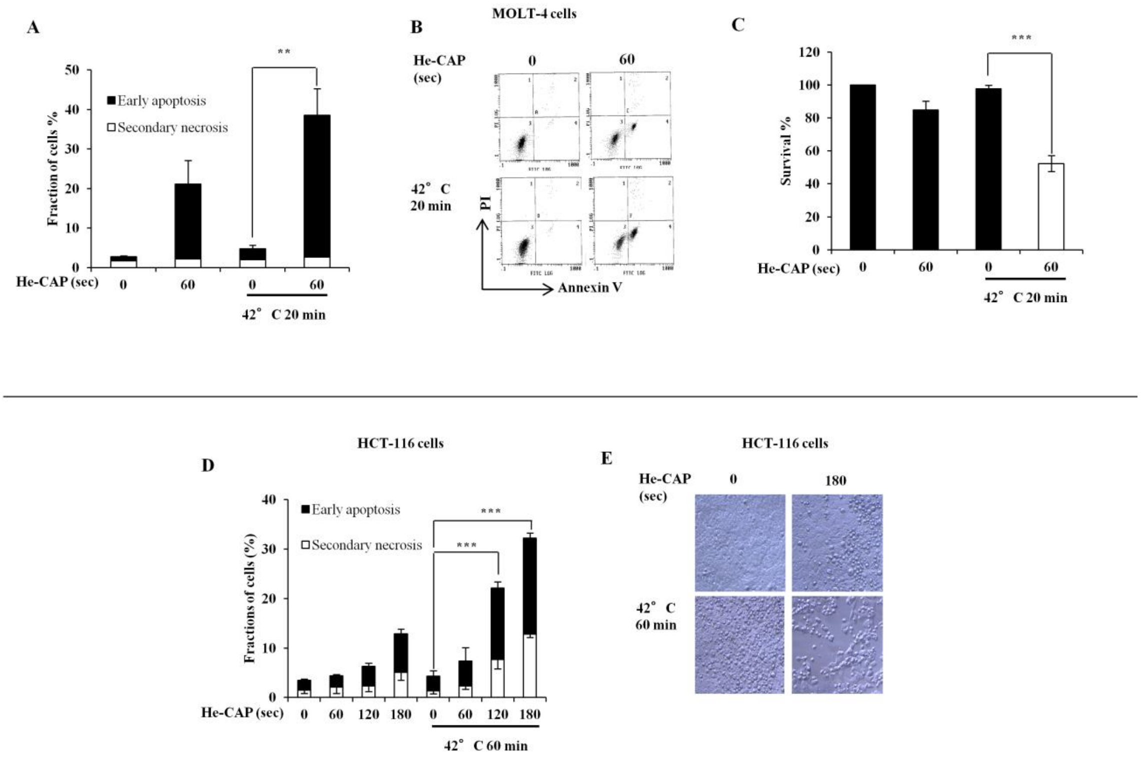

Fig. S2. He-CAP and mild HT induced synergistic effects in other cancer cell lines. (A) Evaluation of apoptosis in MOLT-4 cells. (B) Representative flow cytometric histogram of Annexin V-FITC/PI staining in MOLT-4 cells. (C) Cell survival was assayed 6 h post-treatment following combined treatment by cell counting kit-8. Data are presented as the mean ± SD (n=3). **p<0.01, ***p<0.005, than HT alone. (D) Annexin V-FITC/PI assay was carried out at 24 h following combined treatment in HCT-116 cells. Dose dependent increased in the apoptosis was observed. Data are presented as the mean ± SD (n=3). ***p<0.005, than HT alone (E) DIC live images of HCT-116 cells. Cell proliferation 24 h after combined treatment of He-CAP 180s and HT.

Figure 3. supplementary online

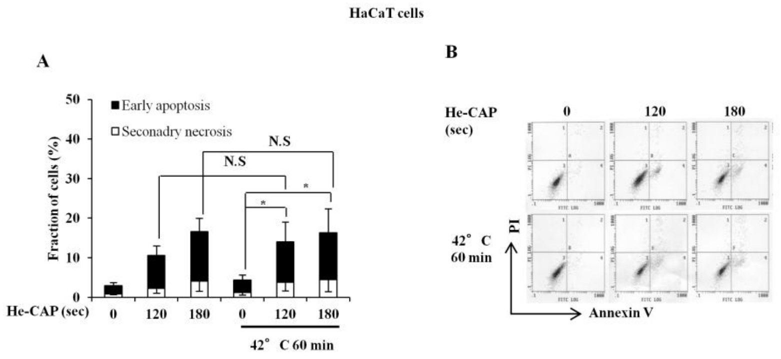

Fig. S3. Effects of combined treatment in human keratinocyte (HaCaT) cell line. (A) Annexin V-FITC/PI assay was carried out at 24 h following combined treatment in HaCaT cells. (B) Representative flow cytometric histogram of Annexin V-FITC/PI staining in HaCaT cells. No significant increased in the apoptosis was observed following combined treatment than He-CAP treatment alone. Data are presented as the mean ± SD (n=4). *p<0.05, than HT alone, N.S (not-significant).

Figure 4. supplementary online

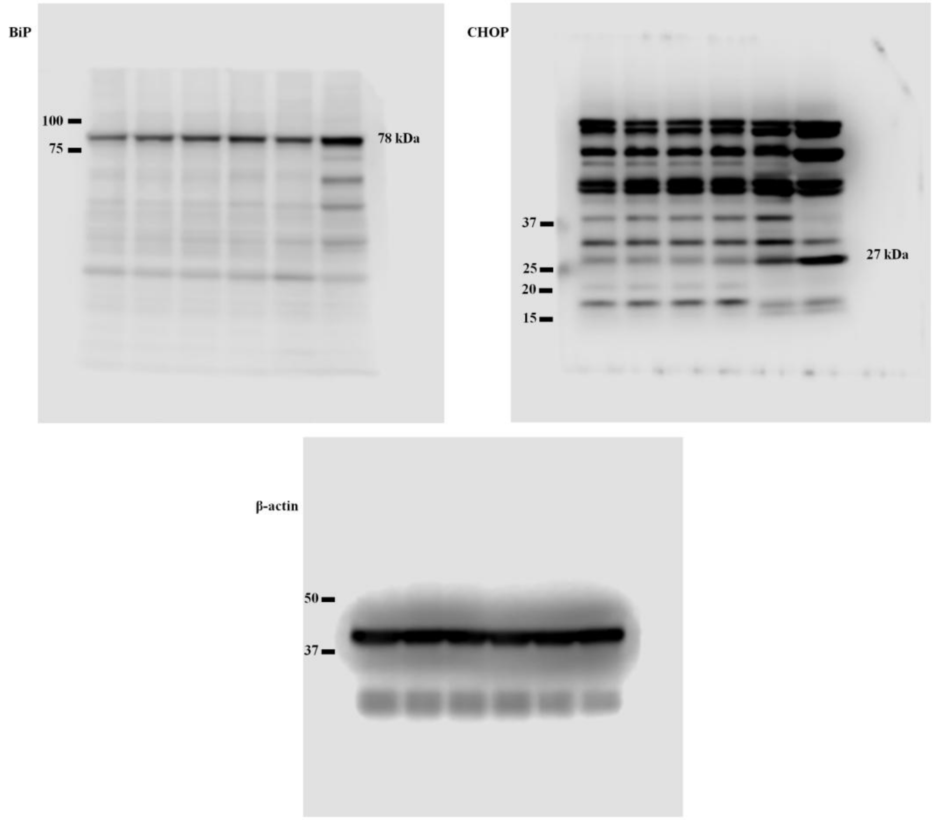

Fig. S4. Full-lengths blots of Figure 3.E.

Figure 5. supplementary online

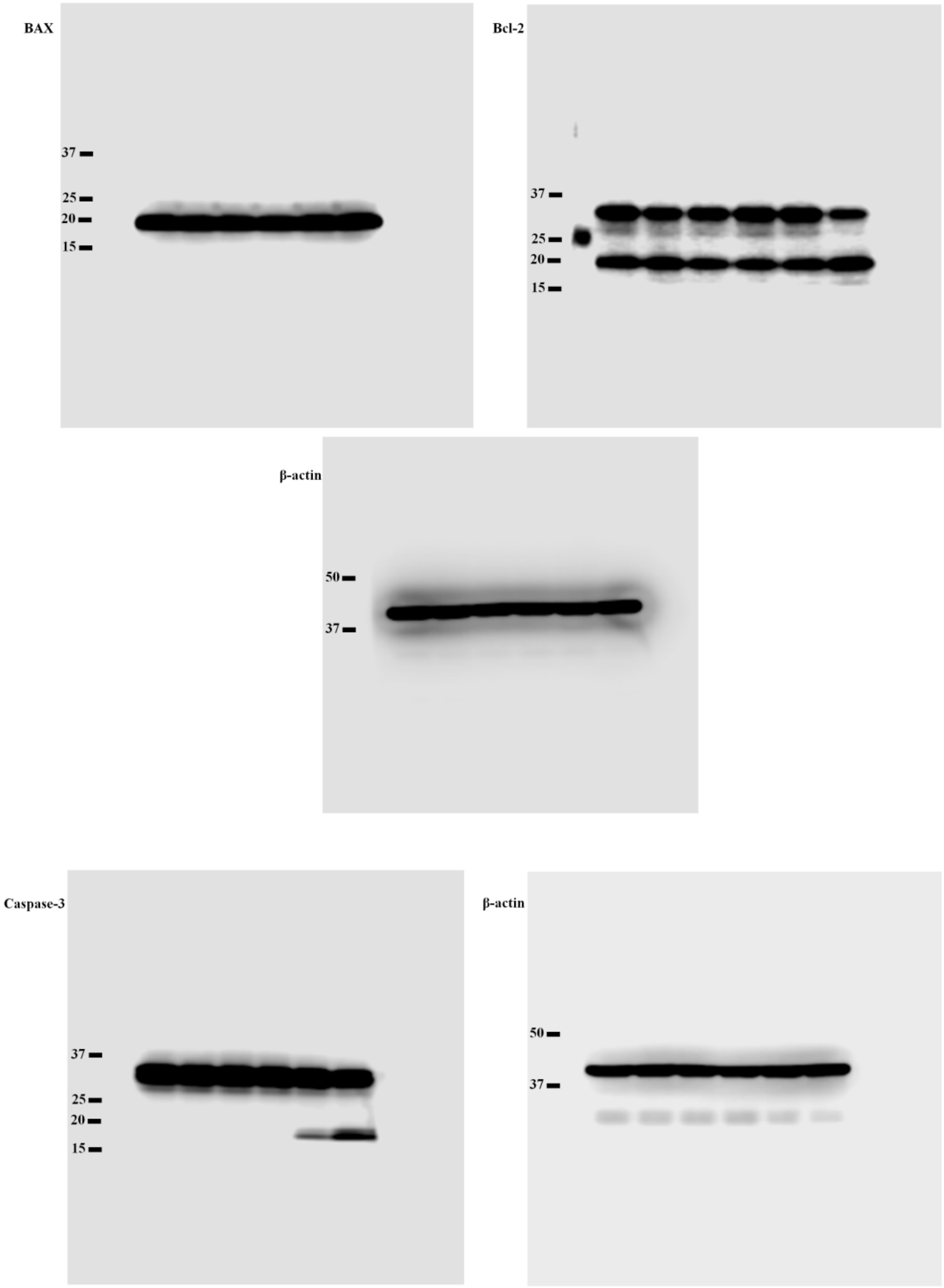

Fig. S5. Full-lengths blots of Figure 5.A.

Figure 6. supplementary online

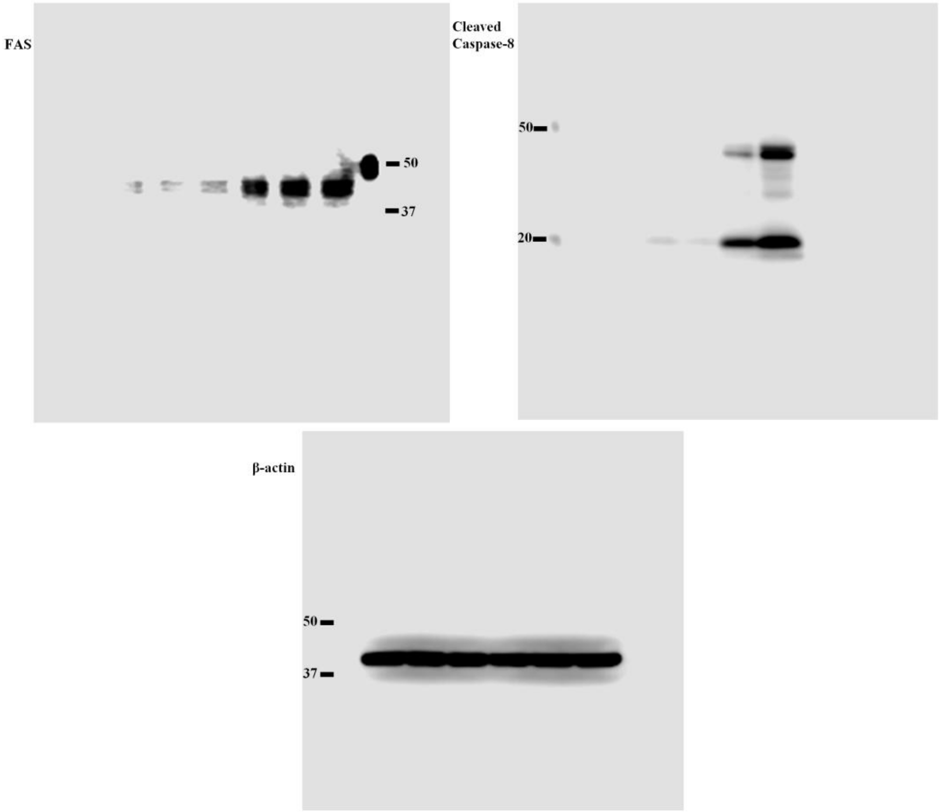

Fig. S6. Full-lengths blots of Figure 5.B.
